# Safety and Effectiveness of Endoscopist-Directed Nurse-Administered Sedation during Gastric Endoscopic Submucosal Dissection

**DOI:** 10.1155/2017/4723626

**Published:** 2017-07-09

**Authors:** Yong Suk Cho, Sang youn Shin, Changhyeok Hwang, Jeonghun Seo, Jong Won Choi, Byung Kyu Park, Sun Young Won, Chun Kyon Lee, Yong Kang Lee, Han Ho Jeon

**Affiliations:** Division of Gastroenterology, Department of Internal Medicine, National Health Insurance Service Ilsan Hospital, Ilsan, Republic of Korea

## Abstract

**Background and Aims:**

Endoscopic submucosal dissection (ESD) is routinely performed in treating gastric neoplasia and requires long-term higher levels of sedation. Endoscopist-directed nurse-administered sedation (EDNAS) has not been well studied in ESD. This study aimed to evaluate the safety and effectiveness of EDNAS for ESD.

**Methods:**

Patients treated with ESD for gastric tumors between 2013 and 2015 were retrospectively collected. Patients were divided into a midazolam-treated group (M group) and a midazolam plus propofol-treated group (MP group). Clinical outcome, safety, effectiveness, adverse events of ESD, and adverse events of sedation were analyzed.

**Results:**

Of 209 collected patients, 83 were in the M group and 126 were in the MP group. Of all patients, 67 patients had the circulatory adverse event during the ESD procedure. Sedation method was the only significant risk factor (M versus MP: 2.17 (1.14–4.15), *p* = 0.019). In analysis of MP subgroups, 47 patients suffered an adverse event from sedation, and current smoking was the only significant association factor for adverse event (0.15 (0.03–0.68), *p* = 0.014).

**Conclusions:**

In performing ESD, the effect of sedation is reduced in smoking patients. EDNAS may be acceptable for ESD under careful monitoring of vital sign and oxygen saturation.

## 1. Introduction

Endoscopic submucosal dissection (ESD) is considered a standard treatment for early gastric neoplasm and is frequently performed in Korea and Japan [1]. ESD is more difficult than the traditional endoscopic mucosal resection (EMR), requires more advanced skills, and takes longer. Therefore, higher level sedation is compulsory during the procedure.

Although a standard sedation method for ESD has not yet determined, benzodiazepine has traditionally been used for endoscopic procedures and propofol is being used more often [2]. Many studies report that propofol is a safer and more efficient sedative drug compared to benzodiazepine [3]. Propofol has an earlier onset and shorter half time than benzodiazepine and is thereby useful for inducing sedation. However, it has a characteristic narrow safety range for cardiopulmonary suppression. For these reasons, there is an ongoing debate regarding whether an anesthesiologist or endoscopist should perform sedation with propofol. To evaluate this issue, there have been many studies performed to determine who should perform sedation and how propofol should be given, with safety and cost-effectiveness taken into consideration.

Despite different sedation methods for endoscopy in different countries, propofol is given by endoscopists in the majority of hospitals in Korea except for few hospitals where it is administered by anesthesiologists. Although endoscopist-directed nurse-administered sedation (EDNAS) is safe, controversy continues around this issue [4]. Many studies have focused on EDNAS safety for routine endoscopic procedures, but few have examined EDNAS for ESD.

The aim of this study was to evaluate the safety and effectiveness of EDNAS for administering midazolam or midazolam plus propofol during ESD for gastric neoplasia.

## 2. Materials and Methods

### 2.1. Patients

In total, 209 gastric neoplasia patients who underwent ESD between March 2013 and October 2015 were included in this study. Patients with an American Society of Anesthesiologists (ASA) [5] physical status class were confirmed and categorized. Data for demographics, ESD results, sedation method, procedure-related adverse events, and adverse events of sedation were retrospectively collected. This retrospective study had an IRB approval.

### 2.2. Strategy for Sedation

As a premedication for all patients, pethidine 25 mg (pethidine HCL, Hana Pharm. Co. Ltd., Seoul, Korea) and the antispasmodic butylscopolamine 20 mg (Freepan, Jeil Pharm. Co. Ltd., Daegu, Korea) were given via intramuscular injection. Patient sedation was performed using the following methods. Midazolam (Bukwang Pharm. Co. Ltd., Seoul, Korea) was given via intravenous injection at an initial dose of 0.05 mg/kg. The sedation level was targeted at 3 or 4 on the Modified Observer's Assessment of Alertness/Sedation (MOAA/S) scale [6]. If the patient became agitated or moving involuntary without any stimulation during the procedure, an additional 1-2 mg of midazolam was given. However, the total amount of midazolam did not exceed 10 mg. If the patient was not adequately sedated with midazolam, an additional 10–20 mg of propofol (propofol, Hana Pharm. Co. Ltd., Seoul, Korea) was given. After propofol injection, neither pethidine nor midazolam was additionally given, and sedation was maintained using only propofol. All injections during the ESD procedure were performed by a trained nurse, and an assessment of sedation was performed by a nurse who had BCLS and sedation education and was not directly involved in the gastric ESD procedure. All patients underwent the ESD procedure in an endoscopy room capable of providing advanced cardiac life support. Patients were given oxygen at 2 L/min through a nasal cannula. Blood pressure was recorded every 5 minutes, and peripheral oxygen saturation and heart rate were continuously monitored via pulse oximetry and electrocardiography. When hypertension (systolic blood pressure ≥ 170 mmHg) persisted despite midazolam or propofol administration, IV nicardipine hydrochloride 1 mg was given. Normal saline fluid infusion was maintained at a rate of 80 mL/hr. If a patient developed desaturation < 90% for longer than 10 sec, supplemental oxygen was increased until the saturation level was above 94%. If the oxygen saturation was not improved within 2 min, the ESD procedure was discontinued to secure the airway by chin lift, jaw thrust maneuver, or mask ventilation. In case of hypotension, we increased the rate of drip or loaded a normal saline (e.g., from 100 to 300 mL).

### 2.3. ESD Procedure

ESD was performed by a team composed of an endoscopist and three nurses, including a nurse responsible for assessing sedation. All ESD was performed by one endoscopist (Jeon). For most procedures, a single-channel upper gastrointestinal waterjet endoscope (GIF-Q260J; Olympus Co., Tokyo, Japan) was used. If the lesion was not easily accessible, a multiband scope (GIF-2TQ260M; Olympus Co., Tokyo, Japan) was used. The location of the lesion was marked with argon plasma coagulation, and the mixture (10% glycerol, 5% fructose in a normal saline solution mixed with hyaluronic acid) was injected toward the outside of the marked region. Afterward, mucous membranes of the marked region were dissected using a knife (dual and/or insulated-tip knives), and then the submucosal layer was dissected. Hemostatic procedures during ESD were performed using hemostatic forceps (Coagrasper; Olympus), and a high frequency generator unit (VIO300D; Erbe Elektromedizin, Germany) was used during the entire procedure.

### 2.4. Outcome Measurements and Definitions

The macroscopic type and location of gastric tumors were categorized according to the Japanese Gastric Cancer Association classification. En bloc resection is defined as a procedure that does not yield multiple piecemeal resections, but one piece resection. Complete resection is defined as a resection that yields histologically confirmed tumor-free lateral and vertical margins. Patients who underwent multiple resections were excluded from the complete resection group. Procedure time was measured from the point of gastric mucosa marking until completion of lesion resection.

Adverse events were defined as follows. For bleeding, adverse events included cases where further blood transfusion was required during the ESD procedure, clinical symptoms such as melena or hematemesis occurred, and hemoglobin levels dropped 2 g/dL or more after ESD. For perforations, cases included those where mesenteric fat was observed under endoscopy or free air was observed under the chest and abdomen on radiography following the procedure. Patients were defined to have pneumonia if they had post-ESD symptoms of coughing, phlegm, and rales, as well as chest radiography abnormalities, and were given antibiotics.

Sedation-related adverse events were defined as respiration and circulatory events. Respiration adverse events during ESD included cases where oxygen saturation was reduced to below 90% and the patient required a chin lift, jaw thrust maneuver, or mask ventilation. Circulatory adverse events included cases where systolic blood pressure dropped below 80 mmHg or 20% or more of baseline.

### 2.5. Statistical Analysis

Categorical variables were analyzed using chi-square or Fisher's exact test. Continuous variables were analyzed using *t*-test. In order to identify related factors with adverse events, a logistic regression model was used. A *p* value below 0.05 was considered statistically significant, and all statistical analyses were performed using IBM SPSS software version 23 (IBM Corp., Armonk, New York, USA).

## 3. Results

### 3.1. Patient Characteristics

There were a total of 209 patients in this study. Of them, 83 patients were in the M group (sedation with midazolam), and 126 patients were in the MP group (sedation with midazolam plus intermittent propofol injection). There were no significant differences between the two groups for age, gender, smoking, drinking, anticoagulant usage, sedatives and psychological drug usage (including sleeping pills), and ASA physical status. Baseline characteristics are summarized in Table 1.

### 3.2. Characteristics of the Gastric Lesions and ESD Outcomes

Characteristics of the gastric lesions and ESD results are summarized in Table 2. There were no differences between the two groups in terms of the location of the lesion, histology, macroscopic appearance, and presence of ulcers. In the M group, there were 55 (66.3%) adenoma, 26 (31.3%) differentiated carcinoma, and 2 (2.4%) undifferentiated carcinoma cases while in the MP group there were 82 (65.1%) adenomas, 43 (34.1%) differentiated carcinomas, and 1 (0.8%) undifferentiated carcinoma. En bloc resection and complete resection rates were 97.6% (81/83) and 95.2% (79/83) in the M group, respectively, and 96.8% (122/126) and 91.3% (115/126) in the MP group. There were no significant differences between the two groups.

The specimen size of the lesion was bigger in the MP group (M versus MP; 30.9 ± 8.1 and 34.2 ± 10.3 mm, *p* = 0.01). Procedure time also was significantly longer in the MP group (M versus MP; 31.7 ± 15.9 and 44.7 ± 32 min, *p* < 0.001). For adverse events such as bleeding, perforations, and pneumonia, there was no difference between the two groups. Frequency of pain two hours postprocedure was significantly higher in the MP group (M versus MP; 4.8% and 18.3%, *p* = 0.005).

### 3.3. Clinical Factors Associated with Adverse Events due to Sedation during ESD

Adverse events due to sedation during the ESD procedure occurred in 67 patients. There were no cases of respiratory adverse event, which need to secure the airway by chin lift, jaw thrust maneuver, or mask ventilation. There were 67 cases of circulatory adverse event. Nine cases were SBP of <80 mmHg, and the others were dropped 20% or more of baseline. Most hypotension events were temporary and recovered. Normal saline loading was performed in only 3 cases (total loading amount: 200 mL, 300 mL, and 300 mL). All 3 cases of hypotension were corrected by normal saline loading.

In order to identify clinical factors associated with circulatory adverse events, a regression model was utilized (Table 3). According to the univariate analysis result, sedation strategy (M versus MP, *p* = 0.047) and current smoking status (*p* = 0.016) were the associated factors for circulatory adverse events. Multivariate analysis results validated that only sedation strategy (M versus MP: 2.17 (1.14–4.15), *p* = 0.019) was a significant factor. Midazolam plus propofol injection caused more adverse effects than midazolam injection. A regression model was again used within the MP group in order to identify adverse event-associated clinical factors (Table 4). Circulatory adverse event occurred in 47 patients in the MP group. Only current smoking was found to be an adverse event-associated factor (0.15 (0.03–0.68), *p* = 0.014).

## 4. Discussion

In this study, good safety was confirmed for using EDNAS in gastric ESD that requires deep sedation for both the M and MP groups. Of all patients, 39.7% (83/209) completed the ESD procedure without further propofol use, and 60.3% needed additional propofol for deep sedation. Propofol is a safe, effective drug used for rapid induction and recovery for endoscopy. Frequency of propofol use by nonanesthesiologists for gastric endoscopy and other procedures is increasing significantly, and propofol is accepted as an effective and stable sedation method [7, 8]. Nevertheless, the most effective and safe sedation protocol for the ESD procedure has not yet been determined.

There was no difference in age, gender, BMI, and ASA physical status between the M group and MP group in this study. Location of the gastric tumor, macroscopic appearance, and histology were also not different. However, the lesion size was significantly larger and the procedure time was significantly longer in the MP group. These results could be explained by the fact that all ESDs were performed by one endoscopist (Jeon). The endoscopist initially removed small lesions in the antrum or body. After gaining more proficiency in ESD, the endoscopist performed cases of various locations, size, and degree of fibrosis to need the different technical skills of ESD. Therefore, intermittent propofol injections were needed for deep sedation. So the lesion size was larger, and the procedure time was longer in the MP group.

Piecemeal resection makes it difficult to assess tumor extension from the resected tumor piece, and therefore, en bloc resection is mandatory for a successful oncologic outcome. Our overall en bloc resection rate was 97.1%, and this value was comparable to other referral centers. Both en bloc resection rate (97.6% M and 96.8% MP) and complete resection rate (95.2% M and 91.3% MP) showed no difference between the groups. Occurrence of adverse events such as bleeding, perforation, and pneumonia was not different between the two groups. Pain occurrence rate two hours postprocedure (4.8% M and 18.3% MP, *p* = 0.005) was significantly higher in the MP group. Previous studies indicated that pain after ESD results from transmural burn or air leak [9–11]. Lee et al. reported that the rate of pain occurrence with fever was 7%, and risk factors were the size and location of the lesion and procedure time [9]. In this study, the MP group had significantly higher frequency of pain occurrence two hours postprocedure, and this is likely due to larger lesion size and longer procedure time for the MP group compared with the M group.

This study showed that additional use of propofol is a risk factor for circulatory adverse events from sedation during the ESD procedure. Recent studies about the use of propofol during ESD reported that adverse events were mild or transient [3, 8, 12]. Propofol has a relatively narrow safety range for cardiorespiratory suppression. It reduces both systemic vascular resistance and cardiac contractility, resulting in reduced cardiac output and suppressed respiration without changes in heart rate [13, 14]. In our study, circulatory adverse events were mild or transient as well.

In order to identify risk factors for circulatory adverse events from sedation using propofol, subgroup analysis was performed in the MP group. The only risk factor identified was smoking. Previous studies have shown that smokers had higher resistance to benzodiazepine sedation than nonsmokers [15]. The sedative effect of benzodiazepines is controlled by the gamma-aminobutyric acid A (GABA_A_) receptor [16]. Chronic exposure to nicotine not only increases GABAergic transmission to the brain but also induces upregulation and desensitization of nicotinic acetylcholine receptors [17]. This process can change the potency of anesthetic drugs that act on GABA receptors. Propofol is also often metabolized in the liver, and its hypnotic effect is controlled by gamma-aminobutyric acid A (GABA_A_) receptor [18]. The hypnotic efficacy of sedation is reduced in smokers compared to non- or ex-smokers. This alteration likely resulted in the lower frequency of circulatory adverse events in smokers during ESD.

Elderly patients are generally defined as subjects who are 75 years or older. The number of elderly people with gastric neoplasms is increasing, but the clinical outcome of gastric ESD for elderly patients with gastric neoplasm remains unclear. Several studies reported and compared the clinical outcomes of elderly patients with nonelderly patients who were treated with ESD. Some studies showed equivalent clinical outcomes and complications between the two groups [19, 20]. However, other studies have shown that it was not [21, 22]. Sedation-related adverse events during ESD were rarely reported in the two groups. Our study also showed that elderly patient (age ≥ 75 yr) was not a clinical factor associated with sedation-related adverse events during ESD.

The clinical importance of this study is the evaluation of trained nurses giving propofol through intravenous injection following directions from the endoscopist. Recent large-scale studies reported the effectiveness of nurse-administered propofol sedation (NAPS) [4, 23, 24]. Injection of propofol by an anesthesiologist is expensive [4], and there are not enough anesthesiologists to provide sedation services for gastric ESD procedures due to increasing demand. The results from this study validated that injection of propofol by trained nurses might be acceptable. Despite the fact that there were no major complications in this study, EDNAS remains controversial and we cannot completely disregard the potential risk of complications from EDNAS during ESD.

There are few limitations to this study. First, it was retrospective, single operator, and a single-center analysis with small sample. Second, this study could not compare continuous propofol infusion and intermittent propofol injection. There is a need for large-scale, prospective, randomized studies to help establish and standardize sedation protocols for future ESD procedures.

## 5. Conclusion

In conclusion, the present study provides insight into the effect that smoking has on sedation. Further, the role of EDNAS was evaluated. We thought that EDNAS might be acceptable for sedation method during gastric ESD.

## Figures and Tables

**Table 1:** Clinical characteristics of the patients.

	M group (*n* = 83)	MP group (*n* = 126)	*p* value
Age (yr)	68 ± 10.2	66.2 ± 10.0	0.206
Gender, *n* (%)			0.458
Male	53 (63.9)	74 (58.7)	
Female	30 (36.1)	52 (41.3)	
Body mass index (kg/m^2^)	24.6 ± 3.8	24.9 ± 3.0	0.619
Smoking history, *n* (%)			0.229
Nonsmoker	63 (75.9)	95 (75.4)	
Ex-smoker	12 (14.5)	11 (8.7)	
Current smoker	8 (9.6)	20 (15.9)	
Alcohol abuse, *n* (%)	11 (13.3)	15 (11.9)	0.773
Use of antiplatelet agents, *n* (%)^a^	22 (26.5)	22 (17.5)	0.117
Regular use of sedatives or psychotrophic drugs, *n* (%)	4 (4.8)	4 (3.2)	0.544
Midazolam dose, mg	5.8 ± 1.9	5.5 ± 1.6	0.228
Propofol dose, mg	—	91.5 ± 72.9	
Antihypertensive agent administration, *n* (%)	1 (1.2)	4 (3.2)	0.65
ASA physical status, *n* (%)			0.692
1	20 (24.1)	37 (29.4)	
2	43 (51.8)	62 (49.2)	
3	20 (24.1)	27 (21.4)	

Values are mean ± SD or *n* (%) of patients. ^a^Antiplatelet agents include aspirin, nonsteroidal anti-inflammatory drugs, and plavix. These medications were discontinued in all patients prior to endoscopic submucosal dissection. SD: standard deviation; M: sedation with midazolam; MP: sedation with midazolam plus intermittent propofol injection; ASA: American Society of Anesthesiologists.

**Table 2:** Characteristics of gastric lesions and outcomes of endoscopic submucosal dissection.

	M group	MP group	*p* value
Number of lesions	83	126	
Location, *n* (%)			0.429
Upper third	4 (3.8)	12 (9.5)	
Middle third	17 (20.5)	27 (21.4)	
Lower third	62 (74.7)	87 (69.1)	
Histology, *n* (%)			0.595
Adenoma	55 (66.3)	82 (65.1)	
Differentiated cancer	26 (31.3)	43 (34.1)	
Undifferentiated cancer	2 (2.4)	1 (0.8)	
Macroscopic appearance, *n* (%)			0.131
Elevated	64 (77.1)	85 (67.5)	
Flat or depressed	19 (22.9)	41 (32.5)	
Ulcer findings of lesions, *n* (%)	0 (0.0)	3 (2.4)	0.157
Specimen size, mm, mean ± SD	30.9 ± 8.1	34.2 ± 10.3	0.01
Outcome of ESD, *n* (%)			
En bloc resection	81 (97.6)	122 (96.8)	0.746
Complete resection	79 (95.2)	115 (91.3)	0.284
Procedure time (min)	31.7 ± 15.9	44.7 ± 32	<0.001
Adverse events of ESD, *n* (%)			
Post-ESD bleeding	1 (1.2)	5 (4.0)	0.406
Perforation	0 (0.0)	1 (0.8)	1.00
Pneumonia	0 (0.0)	1 (0.8)	1.00
Patients' pain (VAS 0–10), *n* (%)			
After 2 hr (VAS > 3)	4 (4.8)	23 (18.3)	0.005

Values are mean ± SD or *n* (%) of patients. SD: standard deviation; M: sedation with midazolam; MP: sedation with midazolam plus intermittent propofol injection; ASA: American Society of Anesthesiologists; VAS: visual analog scale.

**Table 3:** Comparison of clinical factors in circulatory adverse event due to sedation during endoscopic submucosal dissection.

	Nonadverse event (*n* = 142)	Adverse event (*n* = 67)	*p* value		Odds ratio (95% CI)
			Univariate	Multivariate	
Gender, *n* (%)			0.411	—	—
Male/female	89 (62.7)/53 (37.3)	38 (56.7)/29 (43.3)			
Age ≥ 75 (yr)	32 (22.5)	18 (26.9)	0.494	—	—
Sedation method, *n* (%)			0.047	0.019	2.17 (1.14–4.15)
M/MP	63 (44.4)/79 (55.6)	20 (29.9)/47 (70.1)			
Body mass index (kg/m^2^)	24.8 ± 3.6	24.8 ± 2.8	0.93	—	—
Smoking history, *n* (%)	—	—	0.016	0.432	0.42 (0.05–3.65)
Non- or ex-smoker	117 (82.4)	64 (95.5)			
Current smoker	25 (17.6)	3 (4.5)			
Alcohol abuse, *n* (%)	19 (13.4)	7 (10.4)	0.55	—	—
Regular use of sedatives or psychotrophic drugs, *n* (%)	7 (4.9)	1 (1.5)	0.254	—	—
Midazolam (mg)	5.7 ± 1.9	5.6 ± 1.4	0.742	—	—
Propofol (mg)	50.8 ± 66.2	64.5 ± 83.1	0.201	—	—
Procedure time (min)	38.5 ± 25.0	41.7 ± 32.7	0.396	—	—
ASA physical status, *n* (%)			0.981	—	—
1/2	110 (77.5)	52 (77.6)			
3	32 (22.5)	15 (22.4)			

Values are mean ± SD or *n* (%) of patients. SD: standard deviation; M: sedation with midazolam; MP: sedation with midazolam plus intermittent propofol injection; ASA: American Society of Anesthesiologists.

**Table 4:** Comparison of clinical factors in circulatory adverse event due to sedation during endoscopic submucosal dissection in the MP group.

	Nonadverse event (*n* = 79)	Adverse event (*n* = 47)	*p* value	Odds ratio (95% CI)
			Univariate	
Gender, *n* (%)			0.179	—
Male/female	50 (63.3)/29 (36.7)	23 (48.9)/24 (51.1)		
Age ≥ 75 (yr)	15 (19.0)	13 (27.7)	0.26	—
Body mass index (kg/m^2^)	24.9 ± 3.2	24.9 ± 2.7	0.916	—
Smoking history, *n* (%)			0.014	0.15 (0.03–0.68)
Non- or ex-smoker	61 (77.2)	45 (95.7)		
Current smoker	18 (22.8)	2 (4.3)		
Alcohol abuse, *n* (%)	10 (12.7)	5 (10.6)	0.735	—
Regular use of sedatives or psychotrophic drugs, *n* (%)	3 (3.8)	1 (2.1)	0.61	—
Midazolam (mg)	5.6 ± 1.7	5.5 ± 1.5	0.735	—
Propofol (mg)	91.3 ± 64.7	91.9 ± 85.6	0.961	—
Procedure time (min)	43.9 ± 29.5	46.0 ± 36.3	0.716	
ASA physical status, *n* (%)			0.631	—
1/2	61 (77.2)	38 (80.9)		
3	18 (22.8)	9 (19.1)		

Values are mean ± SD or *n* (%) of patients. SD: standard deviation; MP: sedation with midazolam plus intermittent propofol injection; ASA: American Society of Anesthesiologists.
